# RNA-seq-Based Gene Annotation and Comparative Genomics of Four Fungal Grass Pathogens in the Genus *Zymoseptoria* Identify Novel Orphan Genes and Species-Specific Invasions of Transposable Elements

**DOI:** 10.1534/g3.115.017731

**Published:** 2015-04-27

**Authors:** Jonathan Grandaubert, Amitava Bhattacharyya, Eva H. Stukenbrock

**Affiliations:** Max Planck Institute for Evolutionary Biology, August-Thienemann-Str. 2, 24306 Plön, Germany and Christian-Albrechts University of Kiel, Am Botanischen Garten 9-11, 24118 Kiel, Germany

**Keywords:** gene annotation, transposable elements, comparative genomics, species-specific genes

## Abstract

The fungal pathogen *Zymoseptoria tritici* (synonym *Mycosphaerella graminicola*) is a prominent pathogen of wheat. The reference genome of the isolate IPO323 is one of the best-assembled eukaryotic genomes and encodes more than 10,000 predicted genes. However, a large proportion of the previously annotated gene models are incomplete, with either no start or no stop codons. The availability of RNA-seq data allows better predictions of gene structure. We here used two different RNA-seq datasets, *de novo* transcriptome assemblies, homology-based comparisons, and trained *ab initio* gene callers to generate a new gene annotation of *Z. tritici* IPO323. The annotation pipeline was also applied to re-sequenced genomes of three closely related species of *Z. tritici*: *Z. pseudotritici*, *Z. ardabiliae*, and *Z. brevis*. Comparative analyses of the predicted gene models using the four *Zymoseptoria* species revealed sets of species-specific orphan genes enriched with putative pathogenicity-related genes encoding small secreted proteins that may play essential roles in virulence and host specificity. *De novo* repeat identification allowed us to show that few families of transposable elements are shared between *Zymoseptoria* species while we observe many species-specific invasions and expansions. The annotation data presented here provide a high-quality resource for future studies of *Z. tritici* and its sister species and provide detailed insight into gene and genome evolution of fungal plant pathogens.

The genus *Zymoseptoria* (class *Dothideomycetes*, order *Capnodiales*) comprises a group of plant pathogens that infect a variety of *Poaceae* species. The four pathogen species Z. *tritici*, *Z. pseudotritici*, *Z. ardabiliae*, and *Z. brevis* originated in the Middle East and have diverged recently (within the past ∼22,000 years) (Stukenbrock *et al.* 2007). Speciation in the genus *Zymoseptoria* has been associated with specialization and adaptation to different hosts in different environments (Quaedvlieg *et al.* 2011; Stukenbrock *et al.* 2011). Most notably, *Z. tritici* co-evolved and spread with domesticated *Triticum* species and is today a prominent pathogen of wheat worldwide (Banke and McDonald 2005). The poorly described *Z. brevis* species (Quaedvlieg *et al.* 2011) was isolated in Iran like *Z. pseudotritici* and *Z. ardabiliae*, but from another grass host, *Phalaris minor*. Phylogenetic analyses based on information from six DNA loci place *Z. brevis* next to *Z. pseudotritici*, suggesting that this species also shares recent common ancestry with the wheat pathogen (Stukenbrock *et al.* 2012). The close relatedness of the four *Zymoseptoria* species makes this a unique model system to study host specialization and speciation of fungal plant pathogens.

The genome of *Z. tritici* (isolate IPO323) was sequenced and annotated by the Joint Genome Institute (JGI) and provides one of the best-assembled eukaryotic genomes with 21 complete chromosomes sequenced from telomere to telomere (Goodwin *et al.* 2011). Of the 21 chromosomes, eight are considered accessory chromosomes because they can be lost during meiosis without any apparent effect on fitness in sexual progenies (Wittenberg *et al.* 2009). The functional relevance of these accessory chromosomes is so far not known. They contain a higher proportion of repetitive DNA, encode fewer genes (Goodwin *et al.* 2011), and show a dramatically lower level of transcription compared to the core chromosomes (Kellner *et al.* 2014). The previously generated annotation predicted more than 10,000 genes in the genome of IPO323. The pipeline applied for the annotation used several gene predictors including putative full-length genes from EST cluster consensus sequences (Goodwin *et al.* 2011). Nevertheless, this gene annotation comprises many incomplete gene models (14%) without start and/or stop codons.

Different aspects of genome evolution in *Z. tritici* have been studied using comparative analyses of different *Zymoseptoria* genomes (Stukenbrock *et al.* 2010, 2011). A comparative genome study of the reference isolate IPO323 and an isolate of *Z. pseudotritici* allowed a detailed analysis of genome divergence in coding and noncoding sequences and a comparison of chromosome content and synteny in the two species (Stukenbrock *et al.* 2010). Multiple genomes were analyzed in a comparative population genomic analysis including *Z. tritici*, *Z. pseudotritici*, and *Z. ardabiliae* to further assess signatures of positive selection and the speciation history of the three species (Stukenbrock *et al.* 2011). Both studies relied on the previously generated annotation of the *Z. tritici* reference isolate IPO323, and thereby only described evolution of gene content shared between species.

Repeats have also been identified in the reference genome o*f Z. triti*ci (Dhillon *et al.* 2010; Goodwin *et al.* 2011; Ohm *et al.* 2012), but they were only exhaustively classified into transposable element (TE) families recently (Dhillon *et al.* 2014). In fungal genomes, as in many eukaryotic genomes, TEs can constitute a large fraction of the genome, *e.g.*, 40% of the *Pseudocercospora fijiensis* genome (Ohm *et al.* 2012) and 64% of the *Blumeria graminis* genome (Spanu *et al.* 2010). TEs play an important role in the evolution of genomes, and in fungi they are shown to be responsible for large genomic rearrangements (Croll *et al.* 2013; de Jonge *et al.* 2013; Grandaubert *et al.* 2014) and for the creation of highly dynamic regions that provide accelerated diversification and chromatin-based regulation for genes within or near these regions (Rouxel *et al.* 2011; Soyer *et al.* 2014). An in-depth characterization of the complete TE repertoire of the four known *Zymoseptoria* species would allow detailed insights into the impact of repetitive DNA on the evolution of core as well as accessory chromosomes in these pathogens.

We set out to conduct a thorough annotation of protein-encoding genes and repetitive sequences in all four *Zymoseptoria* species. Our goal was to improve the existing annotation of *Z. tritici* and to generate a complete set of gene models and repeat families for the four *Zymoseptoria* species for comparative genome analyses. All predicted gene models were *in silico* functionally annotated. We particularly focused on the presence and distribution of small proteins with signal peptides because these could be effector proteins produced to overcome host immune defenses (de Jonge *et al.* 2011). To characterize a set of core *Zymoseptoria* genes and to identify species-specific orphan genes, we conducted a detailed analyses of gene homologies. Furthermore, we addressed the distribution of TEs in the *Zymoseptoria* genomes with a particular focus on TE content of core and accessory chromosomes of *Z. tritici* and the distribution of TE families between the four *Zymoseptoria* species. The dataset presented here provides not only a new high-quality annotation of *Z. tritici* but also a detailed characterization of gene families among Z. *tritici*, *Z. pseudotritici*, *Z. ardabiliae*, and *Z. brevis*.

## Materials and Methods

### Genome data

For the gene annotation, the genomes of *Z. tritici* (isolate IPO323) (Goodwin *et al.* 2011), *Z. pseudotritici* (isolate ST04IR_2.2.1), and *Z. ardabiliae* (isolate ST04IR_1.1.1) (Stukenbrock *et al.* 2011) were used. For the annotation of *Z. brevis*, the genome of the isolate Zb18110 was sequenced. Genomic DNA was isolated using a standard phenol-chloroform protocol (Sambrook and Russell 2001), and sequencing of 100-bp paired-end reads was performed using a HiSeq2000 Illumina platform (AROS Applied Biotechnology, Denmark). A *de novo* assembly of the *Z. brevis* Illumina reads was generated using the CLC Genomics Workbench version 5 (CLC, Aarhus, Denmark) with standard settings for paired-end read assembly. This assembly is available under the NCBI BioProject (PRJNA273516) on Genbank. The assembled genomes of *Z. pseudotritici*, *Z. ardabiliae* (Stukenbrock *et al.* 2011), and *Z. brevis* used for the gene annotation contained a very low number of repetitive DNA. Therefore, new assemblies better representing the repeat content of these species genomes were constructed using Illumina sequences obtained from the isolates *Z. pseudotritici* ST04IR_5.5, *Z. ardabiliae* ST11IR_6.1.1, and *Z. brevis* Zb163. Assemblies of these new Illumina genomes were generated using SOAPdenovo2 (Luo *et al.* 2012) with optimized k-mer values allowing the inclusion of repeats in the assemblies. These three assemblies are available under the NCBI BioProject (PRJNA274679) on Genbank.

### RNA-seq data

Two previously published RNA-seq datasets were used for *de novo* transcript assembly of *Z. tritici* IPO323 including one dataset obtained from RNA extracted from infected *Triticum aestivum* (cultivar Obelisk) seedlings and one dataset obtained from axenically grown fungal cells (Kellner *et al.* 2014). For *Z. pseudotritici*, *Z. ardabiliae*, and *Z. brevis*, RNA-seq data were obtained from axenically grown cultures. Total RNA was extracted from fungal cells grown in YMS (4 g yeast extract, 4 g malt extract, 4 g sucrose, 20 g bacto agar, 1 liter H_2_O) agar in a shaking incubator at 200 rpm at 18° using the TRIZOL reagent (Invitrogen, Darmstadt, Germany) following the protocol of the manufacturer. Illumina RNA-seq libraries for two axenic culture replicates per species were prepared from an input of 10 µg total purified polyA RNA (Palma-Guerrero *et al.* 2013). Libraries were quantified by fluorometry, immobilized, and processed onto a flow cell with a cBot (Illumina), followed by sequencing-by-synthesis with TruSeq v3 chemistry on a HiSeq2000 at the Max Planck Genome Center (Cologne, Germany). RNA-seq data for *Z. pseudotritici*, *Z. ardabiliae*, and *Z. brevis* are respectively available in the NCBI BioProjects (PRJNA277173, PRJNA277174, PRJNA277175). For data processing, the sequence read quality was first evaluated using FASTQC (www.bioinformatics.babraham.ac.uk/projects/fastqc/). Subsequently, reads were filtered using tools from the Galaxy server, including grooming, trimming, filtering, and masking steps (Giardine *et al.* 2005). Reads with an overall quality score less than 20 were removed. For the remaining reads, all nucleotides with a quality score less than 20 were masked with Ns. For *Z. tritici*, reads from the host (*T. aestivum*) transcriptome were initially filtered out using fastq_screen v0.4.1 (www.bioinformatics.babraham.ac.uk/projects/fastq_screen) as described by Kellner *et al.* (2014).

### Gene annotation

Protein-coding genes were identified using the Fungal Genome Annotation pipeline described by Haas *et al.* (2011). The individual steps of the pipeline are described below.

#### Transcript reconstruction:

Transcript reconstruction using Trinity (Grabherr *et al.* 2011; Haas *et al.* 2013) was carried using *de novo* and genome-guided methods. By the *de novo* method, RNA-seq reads were first assembled into unique sequences of transcripts (contigs) using the Inchworm module within Trinity. Contigs were clustered by the Chrysalis module, and corresponding De Bruijn graphs that represent the possible different isoforms were constructed. In the final step, De Bruijn graphs were processed by the Butterfly module to produce full-length transcripts. In the genome-guided method, RNA-seq reads were first aligned to the genome using GMAP (Wu and Watanabe 2005). Based on these aligned-read clusters, the Chrysalis and Butterfly modules were consecutively executed to produce the final reconstructed transcripts. Transcripts generated by these two methods were combined using the “Program to Assemble Spliced Alignments” (PASA) (Haas *et al.* 2003) pipeline to build a complete set of unique transcripts corresponding to gene models.

#### Training:

First, GeneMark-ES (Ter-Hovhannisyan *et al.* 2008) was used for *ab initio* predictions, because its self-training algorithm allowed the identification of high-quality gene models. Next, evidence from homology searches using tBLASTn (e-value cut-off of 1e-10) (Altschul *et al.* 1990) against a nonredundant protein database (UniRef90) (Suzek *et al.* 2007) and from reconstructed transcripts were used to filter the *ab initio* predicted gene models. Only the complete gene models predicted by GeneMark-ES with a support from the homology-based comparison (100% of coverage for each exon) and with the exact same exon–intron boundaries as in the reconstructed transcripts were selected for the training and testing of *ab initio* gene predictors. The 2693 selected gene models were divided into two sets: one for the training (training set) containing 1611 sequences (60%) and one for assessing prediction accuracy (test set) containing 1082 sequences (40%). The evaluation of the training process was performed using Augustus (Stanke and Waack 2003). For all species, better performance was obtained using datasets of *Z. tritici*. We used the *Z. tritici* training set to train the *ab initio* gene callers for all the species.

#### Gene prediction and annotation:

The trained GeneMark.hmm (Lukashin and Borodovsky 1998) and Augustus programs were used to predict gene models. For *Z. tritici*, Fgenesh (Salamov and Solovyev 2000) gene models obtained from the first annotation by JGI (Goodwin *et al.* 2011) were also included. Gene models obtained from the trained gene predictors were evaluated and combined using the EVidenceModeler (EVM) software (Haas *et al.* 2008) to create a weighted consensus of the gene structures. Weights of 3, 5, and 7 were used for *ab initio* predictions, homology-based predictions, and transcript evidence, respectively. Homology-based gene models obtained from GeneWise (Birney *et al.* 2004) were only used for *Z. tritici* as reliable gene models; they could not be obtained in the much more fragmented genomes of *Z. pseudotritici*, *Z. ardabiliae*, and *Z. brevis*. Based on transcript alignments, the PASA pipeline was used to correct the consensus gene model structure and to resolve conflicts between different isoforms.

### Gene comparison

Based on the four predicted proteomes of the *Zymoseptoria* species, sequence comparisons were performed with BLASTp (e-value cut-off of 1e-5). Obtained pairwise protein alignments were processed using the software SiLiX to build families of homologous proteins (Miele *et al.* 2011). Proteins were clustered together into families if they shared at least 55% of sequence identity over at least 60% of sequence coverage. The building of these families was divided in two steps. First, only complete protein sequences were used to create families. Second, using a semi-bipartite graph, partial protein sequences were added to the existing families.

### Secretome prediction

Plant pathogens secrete proteins to interfere with host immune defenses. Genes encoding secreted proteins are therefore of particular interest due to their potential role in infection and host–pathogen interaction. We screened predicted proteins for the presence of signal peptides and categorized proteins as secreted under the following conditions: (i) if a signal peptide was predicted by both Neural-Network and HMM methods by the software SignalP 3.0 (Emanuelsson *et al.* 2007); (ii) if zero or one transmembrane domain was present in the protein (the domain having at least 30% overlap with the signal peptide) using the software TMHMM 2.0 (Krogh *et al.* 2001); and (iii) if the protein was predicted to be targeted to the secretory pathway by the software TargetP 1.1 (Emanuelsson *et al.* 2007). Secreted proteins with a size of 300 amino acids or less were considered as small secreted proteins (SSPs).

### Functional annotation

Automated functional annotations of the predicted proteins of *Z. tritici*, *Z. pseudotritici*, *Z. ardabiliae*, and *Z. brevis* were performed as previously described by Grandaubert *et al.* (2014). Using a combination of BLAST and InterProScan (Quevillon *et al.* 2005), predicted proteins of each of the four species were categorized into three classes. The first class included proteins with no significant BLAST hit and with no known protein domain identified by InterProScan. These proteins were classified as “predicted proteins,” *i.e.*, predictions with no functional support. This class contained an excess of species-specific genes. The second class, termed “hypothetical proteins,” included proteins with at least one domain identified by InterProScan in the InterPro or Pfam databases (Hunter *et al.* 2012; Coggill *et al.* 2008) or that fulfilled the BLAST result criteria defined by Grandaubert *et al.* (2014) and for which the description indicated “hypothetical protein” in more than 90% of the BLAST hits. Globally, this class contained predictions with poor functional evidence likely corresponding to conserved proteins among several organisms but with no defined functions. The third class included predictions that fulfilled the BLAST result conditions with at least one domain from the InterPro or Pfam database corroborating by the consensus BLAST description. This class was termed the “similar to *function*” and included well-conserved proteins with defined functions in many organisms.

### Repeats and transposable elements identification

Repetitive DNA was identified and annotated in SOAP assembled genomes of the four isolates described above to create a repertoire of repeats specific to the *Zymoseptoria* genus using the REPET pipeline (Flutre *et al.* 2011). To obtain more complete elements, repeat families in each species were clustered using Blastclust from the NCBI-BLAST package (Altschul *et al.* 1990), aligned using Mafft (Katoh and Standley 2013), and new consensuses were then created. These steps were iterated with decreasing values of identity percentage (from 100% to 75%) and coverage (from 100% to 30%) until there was no more clustering of the sequences. Next, the sequences were classified (TEclassifier.py script from REPET) using tBLASTx and BLASTx against the Repbase Update database (Jurka *et al.* 2005) and by the identification of structural features such as long terminal repeats (LTRs) or terminal inverted repeats (TIRs). The sequences were additionally translated into the six reading frames to perform a protein domain search on the conserved domain database (CDD) (Marchler-Bauer *et al.* 2011) using RPS-BLAST. Transposable element (TE) families of each strain were classified and named according to the nomenclature defined by Wicker *et al.* (2007). The 497 repeat families identified in this study are available in FASTA format in Supporting Information, File 1.

## Results

### Improved gene annotation of *Z. tritici*

RNA-seq data and detailed homology searches allowed us to generate an improved annotation of gene models in the IPO323 *Z. tritici* reference genome. Read data obtained from *in planta* and *in vitro* experiments were filtered (Table S1) and used for transcriptome assembly. Assemblies were based on two different but complementary approaches: (i) *de novo*, where the reads were assembled without a reference sequence and (ii) genome-guided, where the IPO323 reference genome was used as a template for transcript assemblies. In total, 68,653 transcripts were generated by the *de novo* method and 73,127 transcripts by the genome-guided method (Table 1). These transcripts were used to create a final database containing 13,847 putative gene models next used for the generation of a training set for *ab initio* predictors and for the correction of predicted gene models. We generated a final training set of 1611 gene models that was used to train tw*o ab initio* gene predictors: Augustus (Stanke and Waack 2003) and GeneMark-hmm (Lukashin and Borodovsky 1998). The outputs of these software were combined with 10,893 predicted gene models of another *ab initio* predictor, Fgenesh (Salamov and Solovyev 2000), obtained from the JGI annotation. We thereby obtained a total of 36,423 different gene models combining models from Augustus, GeneMark-hmm, and Fgenesh. These gene models were processed by EVidence Modeler (EVM) (Haas *et al.* 2008) to identify a consensus model for each gene. Finally, corrections were applied to the gene models using PASA (Haas *et al.* 2003) and the previously reconstructed RNA-seq-based transcripts.

**Table 1:** Annotation features for the four members of the *Zymoseptoria* species complex

	*Z. tritici*		*Z. pseudotritici*	*Z. ardabiliae*	*Z. brevis*
**Assembly**					
Assembly size (Mb)	39.7		32.7	31.5	31.9
No. of scaffolds	21		1164	868	6116
	Previous annotation	New annotation			
**Transcriptome reconstruction**					
No. of transcripts (*de novo*)	—	68,653	16,056	16,353	17,870
No. of transcripts (genome-guided)	—	73,127	19,076	20,193	15,331
No. of gene models	—	13,847	12,027	12,719	10,649
**Gene annotation**					
No. of predicted gene models	10,952	11,839	11,044	10,787	10,557
No. of complete gene models	9397	11,795	10,957	10,686	10,342
No. of partial gene models	1555	44	87	101	215
No. of gene models with RNA-seq support*^a^*	9423	10,048	7618	8297	9939
Average gene length (bp)	1599.8	1620.9	1594.3	1584.9	1592.8
Average transcript length (bp)	1388.8	1462.1	1459.4	1440.9	1462.5
Average protein length (aa)	436.6	487.8	488.0	482.0	487.5
No. of exons	28,309	30,068	26,699	26,231	25,367
Average exon length (bp)	505.7	575.2	604.7	593.7	608.1
Average no. of exons per gene	2.59	2.54	2.42	2.43	2.40
No. of introns	17,357	18,226	15,653	15,445	14,809
Average intron length (bp)	124.1	91.6	90.9	98.4	94.6
Average no. of introns per gene	2.27	2.27	2.16	2.16	2.16
No. of genes with introns	7654	8044	7234	7165	6883
Gene density (genes/Mb)	276.0	298.3	338.2	330.3	331.1
**Functional annotation**					
% of predicted proteins	—	22.9	18.8	19.2	18.1
% of hypothetical proteins	—	31.3	31.2	31.4	32.8
% of *similar to* proteins	—	45.8	50	49.4	49.1
No. of secreted proteins	970*^b^*	874	838	965	700
No. of small secreted proteins (<300 aa)	441*^b^*	441	399	540	331

a Based on alignment with reconstructed transcripts (e-value < 1e-5).

b Extracted from Morais do Amaral *et al.* 2012.

The final gene annotation of IPO323 consists of 11,839 gene models (Table 1). Distributions of the number of gene models along the chromosomes of the annotation presented here and the previous JGI annotation are shown in Figure S1. In our new annotation, only 44 gene models were incomplete (*i.e.*, without start and/or stop codon) compared to 1555 incomplete genes in the previous annotation (Table 1). To identify shared and unique gene models, we used a BLAST search (e-value cut-off of 1e-10). We found 4707 identical gene models between the two annotations. Furthermore, we found 442 gene models uniquely predicted by the JGI pipeline and 1200 models uniquely predicted by the pipeline used here. Gene models of our annotation have an average length of 1621 bp and exhibit longer exons (mean exon length 575 *vs.* 505 bp) and encode longer proteins (488 *vs.* 437 amino acids) when compared to the annotation generated by the JGI pipeline (Table 1). A BLAST comparison at the nucleotide level of our final gene models against the reconstructed transcripts showed that 10,048 out of the 11,839 predicted genes have support based on the RNA sequencing. In the previous annotation, 9423 predicted genes had support compared to the transcripts reconstructed in this study, which underlines the efficiency of this new annotation to predict biologically relevant gene models.

*In silico* functional annotation based on protein signatures and homologies allowed us to assign a function to 43% of the 11,839 predicted proteins. A total of 2714 sequences (22.9%) had no homologies within the NCBI nonredundant protein database and did not include any known protein domain. These represent either species-specific genes of *Z. tritici* or incorrectly predicted genes. Given the fact that 55% of these have RNA-seq support, we considered that the majority of these novel genes must be correctly predicted. Characterization of the *Z. tritici* secretome yielded comparable results to the previously reported *Z. tritici* secretome (Morais Do Amaral *et al.* 2012), representing 874 secreted proteins including 441 small, secreted proteins (SSPs), *i.e.*, with a size inferior to 300 amino acids (Table 1). SSPs of *Z. tritici* have, on average, 2.8-times more cysteine residues compared to the whole proteome (data not shown supporting an extracellular role) (Fass 2012).

### Gene annotation using re-sequenced genomes and RNA-seq data from *Z. pseudotritici*, *Z. ardabiliae*, and *Z. brevis*

RNA-sequencing data obtained from axenic cultures of *Z. pseudotritici*, *Z. ardabiliae*, and *Z. brevis* were filtered (Table S1) and assembled. The resulting transcriptomes include 35,132, 36,546, and 33,201 transcripts clustered into 12,027, 12,719, and 11,542 putative gene models for *Z. pseudotritici*, *Z. ardabiliae*, and *Z. brevis*, respectively (Table 1). The training set established for *Z. tritici* was also for the annotation of the three other *Zymoseptoria* species. For each species, EVM created a weighted consensus of the gene models, which after PASA corrections resulted in 11,044, 10,787, and 10,557 gene models for *Z. pseudotritici*, *Z. ardabiliae*, and *Z. brevis*, respectively (Table 1). The total number of predicted gene models in the re-sequenced *Zymoseptoria* species was similar; however, due to the fragmented genome assemblies of *Z. pseudotritici*, *Z. ardabiliae*, and *Z. brevis*, more partial gene models were predicted compared to models in *Z. tritici* (Table 1). Overall, the gene characteristics of these four species are very similar in accordance with the close phylogenetic relationships. Compared to *Z. tritici*, the functional annotation of the predicted gene models in *Z. pseudotritici*, *Z. ardabiliae*, and *Z. brevis* showed a larger proportion of sequences with an attributed function and a smaller proportion of sequences with no functional evidences. The four annotations are available from http://fungi.ensembl.org/Zymoseptoria_tritici/Info/Index (*Z. tritici*), http://genome.jgi.doe.gov/Zymps1/Zymps1.home.html (*Z. pseudotritici*), http://genome.jgi.doe.gov/Zymar1/Zymar1.home.html (*Z.ardabiliae*), and http://www.uniprot.org/taxonomy/1047168 (*Z. brevis*).

### Gene distribution in *Zymoseptoria* species

**Figure 1:**
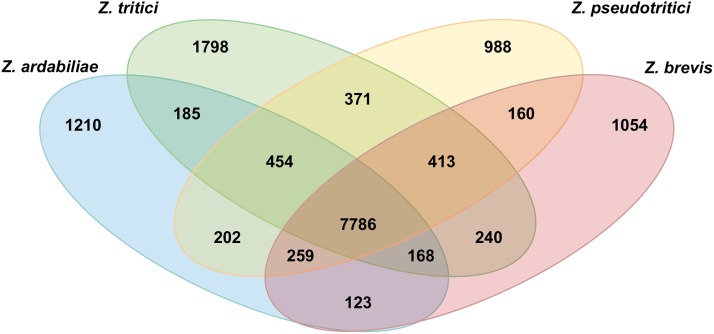
Venn diagram showing the distribution of predicted models in *Z. tritici*: *Z. pseudotritici*, *Z. ardabiliae*, and *Z. brevis*. The categorizations of core *Zymoseptoria* genes, orphan genes, and genes shared by three or two species were performed using a detailed characterization of gene orthology.

Homology relationships among the four *Zymoseptoria* species including orthology (interspecies) and paralogy (intraspecies) were analyzed by a comparison of the predicted proteomes using the software package SiLiX (Miele *et al.* 2011). Of the total 44,227 proteins predicted in the four *Zymoseptoria* species, 39,177 (88.6%) were clustered into 10,612 families. These families belong to three categories. First, there are 10,361 families of orthologous sequences present in at least two species. The different distributions of these families across the *Zymoseptoria* species are shown in Figure 1. Second, there are 236 families of orthologous sequences that also included species-specific paralogous sequences, *i.e.*, independent duplication of a conserved gene occurred in one or more species. Third, there are 15 families of paralogous sequences, *i.e.*, duplication of species-specific gene**s**. The remaining 5050 sequences that could not be classified into a gene family were considered as proteins encoded by orphan genes.

#### Orphan genes:

The genome of *Z. tritici* encodes 1798 orphan genes (15.1% of all the predicted genes), of which 1221 are localized on core chromosomes and 577 on accessory chromosomes. However, accessory chromosomes are significantly enriched in orphan genes because they constitute 79.3% of the genes on these chromosomes compared to 10.9% on core chromosomes (χ^2^ test, *P* < 2.2e-1**6**). A large majority of the orphan genes (95.5%) have no *in silico* attributed function. In general, we could not link species-specific genes to any biological or pathogenicity-related function; however, this uniqueness supports the species-specific nature of the genes that have no homology with known proteins from organisms outside the *Zymoseptoria* species complex. Among the orphan sequences that could be assigned a function, we found predicted functions relating to molecule transport (drugs, proteins, amino acids), primary and secondary metabolism (oxydoreductase activity, nonribosomal peptide synthase), and organic compounds degradation (chitinase, glycoside hydrolase). Small secreted proteins (SSPs) of plant pathogens particularly play an important role as putative effectors during the host infection (Stergiopoulos and De Wit 2009), and we found in *Z. tritici* that SSP-encoding genes were significantly enriched in the orphan gene set compared to the whole proteome: 8% *vs.* 3.7% (χ^2^ test, *P* < 2.2e-16). Orphan genes representing 9–11% of the predicted genes in *Z. pseudotritici*, *Z. ardabiliae*, and *Z. brevis* were likewise significantly enriched in SSP-encoding genes and genes that could not be assigned a function.

#### Paralogous gene**s:**

Families of paralogous genes were only found in *Z. tritici* (eight families) and *Z. ardabiliae* (seven families) and included 23 and 14 genes, respectively. In *Z. ardabiliae*, 57% of these duplicated sequences are SSP-encoding genes, whereas there were no SSP paralogs in *Z. tritici*. In *Z. ardabiliae*, only one family could be associated with a gene function (kinase), whereas in *Z. tritici* several families contained sequences with known protein domains of transposable elements.

#### Orthologous genes:

Families of orthologous genes within the four *Zymoseptoria* species were divided into three categories. The first category comprised the core set of *Zymoseptoria* genes and included 7786 families containing one gene per species (Figure 1). A large proportion of these genes (59.5%) could be associated with a predicted function, whereas 8.3% of them had no homology to genes in other organisms and thereby potentially represented a set of *Zymoseptoria*-specific genes. The second category comprised genes present in three out of the four species and included 1294 families. Genes in this second category appeared to be less conserved because only 33% of them could be associated with function information. Finally, the third category included 1281 families comprising genes only found in two of the four species. Compared to the two other categories, genes with a function represented a smaller fraction (18%).

#### Orthologous genes with paralogy:

A particular category of families was also identified where orthologous sequences were conserved in two or more species with independent duplications in one or more species. This category contained 236 families with 1552 sequences. Most of the families (87%) comprised genes present in the four species. The largest family included 15 genes all encoding an alcohol dehydrogenase with four copies found in *Z. tritici*, *Z. pseudotritici*, and *Z. brevis* and three copies in *Z. ardabiliae*.

### Transposable element annotation

#### TEs in Z. tritici:

**Figure 2:**
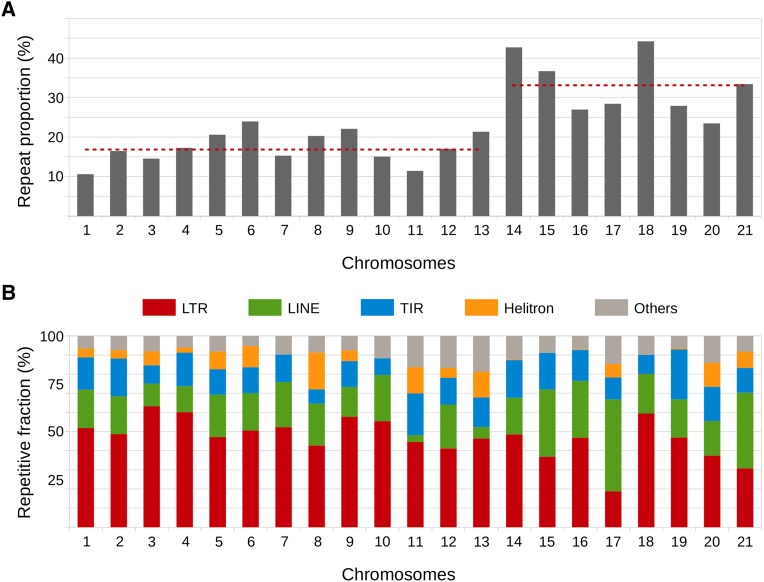
Transposable elements (TEs) characteristics along the 21 chromosomes of *Zymoseptoria tritici*. (A) Proportions of TEs in the chromosomes of *Z. tritici*. The red dashed lines show the mean values of TEs on core (1–13) and accessory chromosomes (14–21). (B) The distribution of five major TE classes across core and accessory chromosomes in *Z. tritici* show a more or less similar distribution and frequency on the two types of chromosomes.

Of the 39.7-Mb genome assembly of *Z. tritici* IPO323, 7.4 Mb (18.6%) **(**Table 2**)** were found to be repetitive DNA, in agreement with the recently reported repeat content of IPO323 (>17%) in Dhillon *et al.* (2014). Repeats were classified into TE families based on features such as terminal repeats, protein domains, and homology with known TE available in the Repbase Update database (Jurka *et al.* 2005). The fact that the assembled genome of *Z. tritici* consists of complete chromosomes allowed a high-quality repeat annotation with only few uncategorized families. TEs of class I (retrotransposons) are the most abundant in the IPO323 genome and represent 13.1% of the total genome size (5.2 Mb), whereas TEs of class II (DNA transposons) represent 4.2% (1.6 Mb) (Table 2). The remaining repetitive sequences represent 0.5 Mb and could not be associated with classified TEs. From the 111 consensus sequences obtained by our procedure (see *Materials and Methods*), we identified 101 families of TEs. The majority of these families belong to the class II (62 families), mainly represented by TIRs (44 families), MITEs (11 families), and Helitron (4 families). Although present in a lower number (32 families), class I elements represent 71% of the repetitive fraction of the genome. The most abundant group of class I elements is the LTR elements from the Gypsy superfamily (14 families), followed by those of the Copia superfamily (12 families) and LINEs (7 families) (Table 2). Interestingly, we also identified one family of a complete tyrosine-recombinase retrotransposon that belongs to the Ngaro superfamily and that otherwise has been described to be absent in *Ascomycota* (Muszewska *et al.* 2013). Distinguishing core and accessory chromosomes of *Z. tritici*, we find that repetitive DNA represents 16.6% of the core chromosomes and 33.6% of the accessory chromosomes (Figure 2A). However, the relative distribution of the major TE families on core and accessory chromosomes does not differ significantly (Wilcoxon test, *P* = 0.3692) (Figure 2B).

**Table 2:** Transposable element content in genomes of the *Zymoseptoria* species complex

		*Z. tritici*			*Z. pseudotritici*			*Z. ardabiliae*			*Z. brevis*		
		Family No.	DNA Amount (kb)	% of Genome	Family No.	DNA Amount (kb)	% of Genome	Family No.	DNA Amount (kb)	% of Genome	Family No.	DNA Amount (kb)	% of Genome
**Class I (retrotransposons)**													
LTR	RLC	12	1017	2.56	6	90	0.23	5	232	0.62	7	827	2.08
	RLG	14	2649	6.67	17	2906	7.50	16	1074	2.88	18	3616	9.09
LINE	RII	4	737	1.86	0	0	0.00	3	76	0.20	4	547	1.38
	RIL	2	747	1.88	0	0	0.00	1	32	0.09	0	0	0.00
	RIX	1	5	0.01	1	83	0.22	1	67	0.18	1	193	0.48
DIRS	RYN	1	23	0.06	0	0	0.00	0	0	0.00	0	0	0.00
SINE	RSX	1	1	0.00	0	0	0.00	0	0	0.00	0	0	0.00
TRIM	RLX-TRIM	4	35	0.09	0	0	0.00	0	0	0.00	1	4	0.01
*Sub-total class I*		*39*	*5214*	*13.14*	*24*	*3080*	*7.94*	*26*	*1481*	*3.97*	*31*	*5186*	*13.04*
**Class II (DNA transposons)**													
TIR	DTT	14	246	0.62	3	51	0.13	4	29	0.08	9	254	0.64
	DTA	7	150	0.38	1	35	0.09	1	4	0.01	5	175	0.44
	DTH	10	184	0.46	0	0	0.00	0	0	0.00	11	292	0.74
	DTM	7	145	0.36	1	44	0.11	0	0	0.00	4	213	0.54
	DTX	6	364	0.92	6	432	1.11	5	76	0.20	3	315	0.79
Unknown	DXX	2	40	0.10	0	0	0.00	0	0	0.00	0	0	0.00
Crypton	DYX	1	6	0.02	0	0	0.00	0	0	0.00	0	0	0.00
Helitron	DHH	4	470	1.18	7	357	0.92	1	39	0.10	5	391	0.98
Maverick	DMM	0	0	0.00	2	476	1.23	0	0	0.00	0	0	0.00
MITE	DTX-MITE	11	48	0.12	1	2	0.01	1	5	0.01	3	11	0.03
*Sub-total class II*		*62*	*1653*	*4.17*	*21*	*1399*	*3.61*	*12*	*153*	*0.41*	*40*	*1652*	*4.15*
**Uncategorized repeats**													
NoCat	NoCat	10	516	1.30	60	1518	3.92	79	1303	3.49	93	2634	6.62
**Total**		**111**	**7383**	**18.60**	**105**	**5997**	**15.47**	**117**	**2937**	**7.87**	**164**	**9472**	**23.87**

As already described by Goodwin *et al.* (2011), repetitive sequences are highly affected by the fungal-specific repeat inactivation mechanism known as repeat-induced point mutations (RIP) (Galagan and Selker 2004). Multiple alignments of several family copies analyzed by RIPCAL (Hane and Oliver 2008) showed an excess of CpA→TpA and TpG→TpA mutations. As hypothesized by Goodwin *et al.* (2011), these mutations should prevent TEs from expressing their own machinery. However, it appears that 98 predicted gene models overlap TE sequences with at least 50% of their length, including 75 genes models that are completely embedded in repetitive DNA. Of the 98 corresponding proteins, 33 harbor domains associated with TEs such as reverse-transcriptase, transposase, or integrase (Table 3). These domains belong to 22 distinct TE families, including 12 TIR, eight LTR, one LINE, and one uncategorized element. Comparison of these gene models with the reconstructed transcripts showed that 48% have RNA-seq support; this suggests that TE families (mainly class II DNA transposons) are still active in the *Z. tritici* genome despite the RIP machinery.

**Table 3:** Gene models with protein domains associated with transposable elements

Protein ID	Length (aa)	Domain Name	Domain Description	Interpro or Pfam Accession No.	Overlapping Repeat Family	RNA-seq Support
Zt09_chr_1_00001	143	RVT_1	Reverse-transcriptase (RNA-dependent DNA polymerase)	PF00078	RIL_element2_ZTIPO323	—
Zt09_chr_1_00017	272	DDE_Tnp_4	DDE superfamily endonuclease	PF13359	DTH_element5_ZTIPO323	—
Zt09_chr_1_01076	477	DDE_3	DDE superfamily endonuclease	PF13358	DTT_element10_ZTIPO323	Yes
Zt09_chr_2_00093	477	DDE_3	DDE superfamily endonuclease	PF13358	DTT_element10_ZTIPO323	—
Zt09_chr_2_00258	409	DDE_Tnp_1_7	Transposase IS4	PF13843	DTX_element1_ZPST04IR55	Yes
Zt09_chr_2_00383	648	DDE_1	DDE superfamily endonuclease	PF03184	DTT_element1_ZTIPO323	Yes
Zt09_chr_2_00481	532	Chromo	Chromo (CHRromatin Organization MOdifier) domain	PF00385	RLG_element5_ZPST04IR55	—
Zt09_chr_2_00619	272	RVT_2	Reverse-transcriptase (RNA-dependent DNA polymerase)	PF07727	RLC_element3_ZTIPO323	—
Zt09_chr_2_00906	219	DDE_1	DDE superfamily endonuclease	PF03184	DTT_element5_ZTIPO323	Yes
Zt09_chr_2_00907	269	DDE_1	DDE superfamily endonuclease	PF03184	DTT_element5_ZTIPO323	Yes
Zt09_chr_3_00204	734	RNaseH-like_dom	Ribonuclease H-like domain	IPR012337	DTA_element2_ZTIPO323	Yes
Zt09_chr_3_00229	349	DDE_3	DDE superfamily endonuclease	PF13358	DTT_element14_ZTIPO323	Yes
Zt09_chr_4_00051	195	DDE_3	DDE superfamily endonuclease	PF13358	DTT_element4_ZB163	—
Zt09_chr_4_00288	371	RNaseH-like_dom	Ribonuclease H-like domain	IPR012337	DTA_element3_ZTIPO323	Yes
Zt09_chr_5_00137	477	DDE_3	DDE superfamily endonuclease	PF13358	DTT_element10_ZTIPO323	Yes
Zt09_chr_7_00478	338	RNaseH-like_dom	Ribonuclease H-like domain	IPR012337	DTA_element6_ZTIPO323	Yes
Zt09_chr_7_00479	108	RNaseH-like_dom	Ribonuclease H-like domain	IPR012337	DTA_element6_ZTIPO323	Yes
Zt09_chr_9_00003	1506	RVT_1	Reverse-transcriptase (RNA-dependent DNA polymerase)	PF00078	RIL_element2_ZTIPO323	—
Zt09_chr_9_00154	621	DDE_1	DDE superfamily endonuclease	PF03184	DTT_element1_ZTIPO323	Yes
Zt09_chr_9_00557	1061	rve	Integrase core domain	PF00665	RLC_element7_ZTIPO323	Yes
Zt09_chr_9_00615	458	DDE_Tnp_1_7	Transposase IS4	PF13843	DTX_element1_ZAST11IR611	Yes
Zt09_chr_10_00001	218	RVT_2	Reverse-transcriptase (RNA-dependent DNA polymerase)	PF07727	RLC_element7_ZTIPO323	—
Zt09_chr_11_00364	162	Chromo	Chromo (CHRromatin Organization MOdifier) domain	PF00385	RLG_element7_ZPST04IR55	—
Zt09_chr_13_00054	869	DDE_3	DDE superfamily endonuclease	PF13358	DTT_element10_ZTIPO323	Yes
Zt09_chr_13_00057	150	RVT_2	Reverse-transcriptase (RNA-dependent DNA polymerase)	PF07727	RLC_element2_ZTIPO323	—
Zt09_chr_13_00271	480	RNaseH-like_dom	Ribonuclease H-like domain	IPR012337	DTA_element1_ZTIPO323	Yes
Zt09_chr_17_00004	876	RVT_1	Reverse-transcriptase (RNA-dependent DNA polymerase)	PF00078	RIL_element2_ZTIPO323	—
Zt09_chr_17_00088	523	RVT_1	Reverse-transcriptase (RNA-dependent DNA polymerase)	PF00078	RIL_element2_ZTIPO323	—
Zt09_chr_18_00008	750	RNaseH-like_dom	Ribonuclease H-like domain	IPR012337	NoCat_element60_ZAST11IR611	—
Zt09_chr_19_00003	169	RVT_2	Reverse-transcriptase (RNA-dependent DNA polymerase)	PF07727	RLC_element5_ZTIPO323	—
Zt09_chr_19_00040	947	RVT_2	Reverse-transcriptase (RNA-dependent DNA polymerase)	PF07727	RLC_element9_ZTIPO323	—
Zt09_chr_19_00041	107	UBN2_3	gag-polypeptide of LTR copia-type	PF14244	RLC_element9_ZTIPO323	—
Zt09_chr_21_00003	1609	RVT_1	Reverse-transcriptase (RNA-dependent DNA polymerase)	PF00078	RLG_element9_ZTIPO323	—

#### TEs in Z. pseudotritici, Z. ardabiliae, and Z. brevis:

To improve the repeat annotation of *Z. pseudotritici*, *Z. ardabiliae*, and *Z. brevis*, we generated improved assemblies of Illumina-sequenced genomes of the isolates: ST04IR55 of *Z. pseudotritici*, ST11IR611 of *Z. ardabiliae*, and Zb163 of *Z. brevis*. The assembly sizes were 38.8 Mb, 37.3 Mb, and 39.8 Mb, respectively, compared to the 39.7 Mb genome assembly of *Z. tritici*.

The three genomes reflect different extents of repeat invasion: repetitive DNA comprises 7.9% of the *Z. ardabiliae* genome, 15.5% of the *Z. pseudotritici* genome, and 23.9% of the *Z. brevis* genome, including a total of 117, 105, and 164 repeat families identified in the genomes, respectively (Table 2). As a result of the fragmented assemblies, the number of uncategorized repeat families in the *Z. pseudotritici*, *Z. ardabiliae*, and *Z. brevis* genomes represent more than 50% of all the families and between 25% and 44% of the repetitive fraction. In contrast, the number of uncategorized families in the *Z. tritici* genome represents only 9% of the families and 7% of the repetitive fraction. Nevertheless, it was possible to classify some TE families of *Z. pseudotritici*, *Z. ardabiliae*, and *Z. brevis* and to record general patterns of TE distribution among the three genomes. As in the *Z. tritici* genome, class I TEs that are mainly represented by LTR-Gypsy elements are most abundant and account for 51–55% of the total repetitive fraction (Table 2). LINE elements are hardly represented in the *Z. pseudotritici*, *Z. ardabiliae*, and *Z. brevis* assemblies; however, this may be an artifact of the fragmented assemblies. In *Z. pseudotritici* and *Z. brevis*, class II TEs are mainly composed of TIRs that represent approximately 20% of the repetitive fraction similar to class II TEs in *Z. tritici*. Helitron elements were also identified in *Z. pseudotritici*, *Z. ardabiliae*, and *Z. brevis*; however, they accounted for only 1–6% of the repetitive fraction compared to 6.4% in *Z. tritici*. Maverick elements (Pritham *et al.* 2007**)** were only identified in *Z. pseudotritici* and were the third most represented TE family in the genome (Table 2). The unique presence and distribution of Maverick elements in *Z. pseudotritici* could result from a recent invasion of the genome followed by massive waves of transposition of these elements.

#### The complete repertoire of repeats in Zymoseptoria spp:

In total, we found 497 repeat families in the four *Zymoseptoria* species, including 120 families of class I TEs (24.1%), 135 families of class II TEs (27.2%), and 242 uncategorized families (48.7%). We could improve the TE annotation using the complete *Zymoseptoria* TE library generated here. We found 19.9% of repeats (+1.3%) in the genome of *Z. tritici*, 17.6% (+2.2%) in the genome of *Z. pseudotritici*, 12.7% (+4.8%) in the genome of *Z. ardabiliae*, and 25.2% (+1.3%) in the genome of *Z. brevis*.

Addressing TE evolution in the *Zymoseptoria* genomes, we found that some families identified independently in each species were derived from a common ancestral TE family. An analysis of the 497 families was performed using Blastclust (Altschul *et al.* 1990) with a coverage cut-off of 50% and an identity percentage cut-off of 80%; this resulted in the clustering of 76 families into 34 larger groups (Table S2). Fourteen of these groups were composed of TIR elements, eight groups of LTR-Gypsy elements, six groups of uncategorized elements, three groups of LTR-Copia elements, two groups of Helitrons, and one group of LINE elements. Only one of these groups included TE families present in each of the four species; this suggests that this element, an LTR-Gypsy element, was present in the genome of the common ancestor of the four species (Table S2). Interestingly, this element may have expanded prior to the divergence of species because we observed a comparable size expansion in the four species comprising between 120 and 180 kb in each genome. Given the phylogenetic tree of the four *Zymoseptoria* species, as shown in Stukenbrock *et al.* (2012), the other TE groups were distributed in either two or three species with eight different ancestral relationships (Table S2). This analysis mainly showed that these species have undergone events of invasion and expansion of distinct TEs after their divergence.

## Discussion

“Omics” analyses such as comparative and evolutionary genomics, transcriptomics, epigenomics, or proteomics mainly rely on the accuracy of predicted gene models. The *Zymoseptoria* species complex provides a unique model system for the application of “omics” data to study the underlying genetics of pathogenicity and host specificity of plant pathogens (Stukenbrock *et al.* 2010, 2011, 2012). However, updated and comparable annotations of gene and TE content in species of *Zymoseptoria* have so far been missing. Here, we present a new gene and repeat annotation of the prominent fungal wheat pathogen *Z. tritici* and its three close relatives: *Z. pseudotritici*, *Z. ardabiliae*, and *Z. brevis*; therefore, we provide a valuable resource for future functional, experimental, and evolutionary studies.

We conducted the re-annotation of the IPO323 genome using a pipeline that identifies the most probable gene models based on information from transcript assemblies, homology searches, and *ab initio* gene predictors (Haas *et al.* 2011). This allowed us to identify 11,839 gene models, including 1200 models that were not predicted previously by the JGI. The support of RNA-seq data for most of these genes underlines the potential of our pipeline for identifying novel genes.

We applied the same annotation pipeline to re-sequenced genomes of the three close relatives of *Z. tritici*, *Z. pseudotritici*, *Z. ardabiliae*, and *Z. brevis*, and could call comparable numbers of genes in these species (*∼*10,500 models). Of these, 7786 represent core *Zymoseptoria* genes shared among the four species. Our exhaustive search for gene orthology also identified genes shared by two or three species and suggested recent gains or losses of genes among the *Zymoseptoria* species. Notably, we also identified 1798 orphan genes in *Z. tritici* enriched in putative pathogenicity-related factors. Species-specific orphan genes in *Z. tritici* and in the three other species may include key determinants of virulence in wheat and other grass hosts. Further functional studies will allow this hypothesis to be tested.

Recently, a new identification and classification of repeats and TEs in *Z. tritici* was conducted (Dhillon *et al.* 2014). In the present study we found very similar patterns in terms of repeat and TE distribution, number of families, and evidence of TE activity. However, we could further improve the assignment of repeat families to known TE families; 39.8% of the 93 repeat families identified by Dhillon *et al.* were not categorized into known TE classes. We were able to improve the TE classification, leaving only 9% of the 111 repeat families unclassified. The TE annotation pipeline used here contains a number of supplementary steps of clustering of the REPET outputs and allowed us to identify more complete elements. These were all manually checked to avoid the generation of false or chimera elements.

The gene and TE annotation of the four *Zymoseptoria* species genomes coupled with preliminary comparative analysis allowed us to highlight interesting features relevant to the biological life traits of these organisms. Establishment of homologous sequence families across the four species showed that a large part of genes are shared between two or more species and that the major difference of gene content relies on orphan genes. These orphan genes, in all four species, are significantly enriched in putative pathogenicity-related genes and potentially play a role in the determination of host specificity. Similar presence–absence patterns of pathogenicity-related genes were shown to be strong determinants of host range in plant pathogens, *e.g.*, between lineages of the rice blast pathogen *Magnaporthe oryzae*, the wilt pathogen *Verticillium dahliae*, and lineages of the *Leptosphaeria maculans–Leptosphaeria biglobosa* species complex (Couch *et al.* 2005; de Jonge *et al.* 2012; Grandaubert *et al.* 2014). Similarly, comparison of TE families showed a majority of species-specific sequences; this underlies the fact that each species genome has been widely invaded after the species divergence. We speculate that TEs may have played a role in host specialization by the acquisition or modification of pathogenicity-related traits. Also, large TE-rich regions could have played a role in speciation through the suppression of homologous recombination in chromosomal regions with inversions or translocations as proposed for the *Leptosphaeria maculans–Leptosphaeria biglobosa* species complex (Grandaubert *et al.* 2014). In Dhillon *et al.* (2014), the authors stated that several genes with putative pathogenicity-related functions were found to be associated with TEs. Preliminary analyses of genes and TEs tend to show that orphan genes are located significantly closer to TEs than other nonorphan genes (J. Grandaubert, unpublished data). TEs thus may play a role in the origin and evolution of these genes.

The annotation data presented here will greatly improve our understanding of gene evolution, adaptation, and the role of TEs in pathogen evolution. The predicted gene models will furthermore greatly support future studies of gene expression in *Z. tritici* and its related species and provide a strong basis to understand the underlying genetics and molecular biology of host–pathogen interactions in *Zymoseptoria* spp.
